# How much do Europeans know about the link between alcohol use and cancer? Results from an online survey in 14 countries

**DOI:** 10.1186/s13104-024-06707-w

**Published:** 2024-02-20

**Authors:** Maria Neufeld, Daša Kokole, Daniela Correia, Carina Ferreira-Borges, Aleksandra Olsen, Alexander Tran, Jürgen Rehm

**Affiliations:** 1 WHO Regional Office for Europe, UN City, Marmorvej 51, 2100 Copenhagen, Denmark; 2 CAPHRI Care and Public Health Research Institute, Maastricht University, 6200 Maastricht, MD POB 616, the Netherlands; 3 EPIUnit - Instituto de Saúde Pública, Universidade do Porto, Porto, Portugal; 4 Research Institute for Mental Health Policy Research, Centre for Addiction and Mental Health, 33 Ursula Franklin Street, M5S 2S1 Toronto, ON Canada; 5 Centre for Addiction and Mental Health, Dalla Lana School of Public Health, Institute of Health Policy, Management and Evaluation, University of Toronto, 155 College Street, Toronto, Canada; 6 University Medical Center Hamburg-Eppendorf (UKE), Martinistraße 52, Hamburg, Germany; 7 Public Health Agency of Catalonia, WHO Collaborating Centre on Substance Use, Noncommunicable Diseases and Policy Impact, 81-95 Roc Boronat St, 08005 Barcelona, Spain

**Keywords:** Alcohol, cancer, Knowledge, Noncommunicable diseases, Prevention, Public health, Risk factors

## Abstract

**Objective:**

In the EU, which has the highest drinking levels worldwide, cancer is the primary cause of alcohol-attributable deaths. Existing studies show gaps in public knowledge, but there is lack of systematic appraisal. The report presents original data from a cross-sectional survey conducted within the framework of an online experimental study in 14 European countries, which among other things assessed baseline knowledge of the alcohol-NCD link, particularly cancer.

**Methods:**

Online questionnaire among adults who consume alcohol conducted in 14 countries in 2022–2023 using different recruitment strategies and applying population weights for the final sample. Baseline assessments measured participants’ knowledge of alcohol-attributable health issues (with a specific focus on cancer).

**Results:**

Baseline knowledge assessment showed that 90% indicated a causal role of alcohol for liver disease, 68% for heart diseases, and only 53% for cancer. Knowledge of specific alcohol-attributable cancer types was lower, with 39% aware of the link between alcohol use and colon cancer, 28% regarding oral cancer, and only 15% regarding female breast cancer. Knowledge levels varied across different countries and population groups.

**Conclusion:**

Most Europeans do not know which cancers can be caused by alcohol use and knowledge is low specifically for female breast cancer. More awareness raising and prevention efforts are needed, such as the placement of cancer-specific health warnings on alcohol container labels.

**Supplementary Information:**

The online version contains supplementary material available at 10.1186/s13104-024-06707-w.

## Introduction

Noncommunicable diseases (NCDs) remain the largest health burden of our time, killing 41 million annually, equivalent to 74% of all deaths globally [1]. Cardiovascular diseases and cancers account for most of the total deaths, with a share of 44% and 23%, respectively. Alcohol use is a major risk factor for NCDs and is a cause for more than 200 diseases and injuries in the International Classification of Diseases [2]. Globally, two-thirds of alcohol-attributable deaths are NCD deaths, and this proportion is even higher in the European Union (EU), where alcohol consumption is the highest globally and where 75% of all alcohol-attributable deaths are due to NCDs, with cancer (29%) being the leading cause [3, 4]. Although “ethanol in alcoholic beverages” has been classified as a group 1 carcinogen by the International Agency for Research on Cancer over three decades ago, this knowledge has still not entered broad public awareness in most countries [5].

The present short report summarizes original data on knowledge of the link between alcohol consumption and various health harms, including cancer, from a large-scale European study. Data were gathered during a baseline assessment module of a larger online experiment, which assessed the impact of different health messages on labels of alcoholic beverages in 14 European countries.

## Main text

### Methods

The study has been conducted as part of a broader project, which focuses on the development of evidence to support the implementation of effective health warnings on labels of alcoholic beverages in the EU [6]. The study involved an online survey conducted in 14 countries with diverse drinking patterns: Austria, Belgium, Estonia, France, Germany, Ireland, Latvia, Lithuania, Netherlands, Norway, Portugal, Slovenia, Spain, and Sweden. The survey was developed in English and underwent a pre-testing phase involving close professional networks of the core team to assess the survey’s comprehensiveness and correct execution, and of the different experimental components of the label designs (see Annex for the English version). After completion of pre-testing, the survey was translated into 12 different languages, with each translation independently checked by a second native speaker, and any discrepancies were discussed by the team. A pre-test was separately conducted for each language version as well, and any identified issues with language or display of the different labels were addressed. Data was collected through two waves in 2022–2023, using different recruitment strategies, namely distribution of the survey link through national agencies and non-governmental organizations, complemented by sponsored Facebook advertising. Post-stratification weighting for age, gender and education attainment was applied using EUROSTAT distributions so the resulting weighted distribution was representative of the population structure of each country [7]. Inclusion criteria for participation were 18 + years of age (except in Lithuania where the minimum legal drinking age is 20+) and having consumed alcohol in last 12 months.

As part of baseline assessment, participants’ knowledge of alcohol-attributable health harms was measured. The knowledge assessment was based on previous studies [8, 9] and included identifying conditions associated with alcohol use, such as cancer, heart disease, liver disease, and respiratory disease. Participants could also select answers such as “don’t know” or “none.” If they selected cancer, they were asked to specify the cancer type caused by alcohol consumption from a list which included female breast cancer, oral cancer, colon, and liver cancers, as well skin cancer as a control condition with no proven link to alcohol use.

More details on the design, sampling, and data processing strategies can be found in the published research protocol and a specific methods paper [6, 10].

The proportion of respondents which identified each condition associated with alcohol use was analysed for the total sample, by country, gender, age, and education level, and significant differences assessed through Chi-squared tests. In addition, multivariate logistic regressions were fitted to each of the outcomes to assess the multivariate effect of the demographic variables, as well as to test interaction effects between them. Significant interaction effects were found between gender and age for all outcomes, as well as between age and education for most outcomes. Since all the interactions detected included the variable age group, we decided to stratify the analysis by this variable. Thus, the multivariate effect of gender and education was estimated through adjusted odds ratios (OR) and the respective 95% confidence intervals (95%CI), stratified by age group (Table A3, supplementary material).

## Results

The final sample size comprised *N* = 19,601 participants, and included only responses from participants who met the outlined inclusion criteria, i.e. who were at least 18 years old (or 20 in the case of Lithuania) and who have consumed alcoholic beverages in the past 12 months. After weighting, the marginal distributions of age, gender, and education matched the nations’ actual distributions for most countries, and the average absolute difference from EUROSTAT data was 2.2% (see Annex Table A1 and A2 for the reported results in the unweighted sample).

**Table 1:** Proportion of respondents selecting conditions where alcohol consumption increases cancer risk, total, and by country, using weighted data^a^

All countries, *N* = 19,601	Total	Austria	Belgium	Estonia	France	Germany	Ireland	Latvia	Lithuania	Netherlands	Norway	Portugal	Slovenia	Spain	Sweden	*p*-value^b^
N	**19,601**	1356	885	969	1876	2565	923	1386	511	758	1126	2345	1144	2826	931	
**Cancer**	**53%**	55%	60%	43%	72%	65%	60%	26%	41%	60%	48%	45%	39%	56%	52%	< 0.001
**Heart disease**	**68%**	70%	76%	77%	82%	77%	68%	69%	73%	67%	56%	50%	56%	66%	68%	< 0.001
**Liver disease**	**90%**	96%	89%	93%	89%	95%	93%	89%	90%	92%	89%	81%	91%	87%	93%	< 0.001
**Respiratory disease**	**11%**	8%	10%	14%	16%	13%	19%	8%	10%	11%	5%	11%	8%	14%	6%	< 0.001
**Don’t know**	**5%**	2%	4%	5%	4%	2%	5%	6%	2%	6%	9%	9%	6%	7%	4%	< 0.001
**None**	**2%**	1%	1%	1%	1%	2%	0%	2%	4%	1%	1%	4%	2%	1%	2%	< 0.001
Female breast cancer*	**15%**	15%	17%	13%	24%	24%	28%	7%	13%	23%	10%	7%	9%	13%	15%	< 0.001
Liver cancer*	**50%**	51%	57%	41%	69%	62%	58%	25%	39%	56%	42%	43%	37%	53%	47%	< 0.001
Colon cancer*	**39%**	44%	47%	29%	55%	53%	44%	20%	28%	46%	34%	23%	30%	39%	38%	< 0.001
Oral cancer*	**28%**	28%	35%	18%	44%	41%	36%	10%	19%	33%	15%	21%	25%	28%	20%	< 0.001
Skin cancer*	**6%**	6%	5%	6%	12%	9%	7%	3%	6%	7%	2%	3%	2%	6%	3%	< 0.001
Don’t know*	**2%**	2%	1%	2%	2%	2%	1%	1%	1%	2%	3%	1%	1%	1%	4%	< 0.001
None*	**0%**	0%	0%	0%	0%	0%	1%	0%	0%	0%	0%	0%	0%	0%	0%	< 0.001

* Only respondents selecting “cancer’ were asked to respond to the question about specific cancers, percentage represents proportion of all respondents

^a^ Weighted by age, gender, and education on country level based on the population distribution of EUROSTAT

^b^ Chi-squared tests for differences in proportions were conducted to assess statistical significance. *P*-values below 0.05 are considered statistically significant

Table 1 presents the main results of the pre-intervention knowledge assessment in the total sample and in country sub-samples, highlighting that while most participants (90%) have correctly associated liver disease with alcohol use, this proportion was considerably lower for heart disease (68%), where only half selected cancer (53%). Among those who selected cancer, and consequently were presented with a list of different types of cancers, the most-selected type of cancer was liver, followed by colon and oral cancers, while a smaller proportion selected female breast cancer. When calculating the response rates as percentages of all respondents to highlight overall knowledge levels of the specific cancers caused by alcohol use, the data highlight that half of all respondents (50%) were able to correctly name liver cancer as a condition associated with alcohol use, 39% identified colon, 28% selected oral cancers, and only 15% associated alcohol use with female breast cancer. Interestingly, 6% indicated skin cancer as being associated with alcohol use. There was also large variation in the responses between countries. Less pronounced cross-country differences were observed in knowledge levels related to liver disease, varying from 96% (Austria) and 81% (Portugal). Knowledge regarding alcohol use and other alcohol-attributable diseases varied even more widely, between 82% (France) and 50% (Portugal), with the largest difference in cancer knowledge observed between France (72%) and Latvia (26%).

Overall, women had higher knowledge in relation to all conditions than men, with the largest difference observed for breast cancer (21% vs. 10%, respectively). Also younger, and better educated participants demonstrated higher knowledge of the various alcohol-attributable conditions (Table 2). However, results show that age modifies the magnitude of differences in knowledge between genders and education levels, despite never inverting the signal (see Annex Table A3 for the adjusted logistic regression results). For example, among young adults between 18 and 34, when compared to men, women had 1.15 (95%CI: 1.03, 1.27) times higher odds of selecting cancer as a condition for which risk increases with alcohol consumption. Also, the difference between men and women increased with age, with women over 55 years old having odds of selecting cancer as a condition for which risk increases with alcohol consumption of 1.46 (95%CI: 1.32, 1.62) times higher than men.

**Table 2:** Proportion of respondents selecting the condition where alcohol consumption increases the risk of, by gender, age, and education, using weighted data^a^

All countries, *N* = 19,601	Gender			Age				Education		
	Women	Men	*p*-value^b^	18–34	35–54	55+	*p*-value^b^	Secondary or less	Tertiary	*p*-value^b^
N	9,583	10,018		5,753	7,975	5,873		12,163	7,438	
**Cancer**	57%	49%	< 0.001	56%	53%	49%	< 0.001	47%	62%	< 0.001
**Heart disease**	71%	64%	< 0.001	74%	68%	61%	< 0.001	64%	73%	< 0.001
**Liver disease**	93%	86%	< 0.001	89%	91%	89%	< 0.001	88%	93%	< 0.001
**Respiratory disease**	12%	11%	< 0.001	15%	11%	9%	< 0.001	10%	13%	< 0.001
**Don’t know**	4%	7%	< 0.001	6%	5%	6%	0.001	6%	3%	< 0.001
**None**	1%	3%	< 0.001	1%	1%	3%	< 0.001	2%	1%	0.098
Female breast cancer*	21%	10%	< 0.001	15%	16%	14%	0.004	10%	23%	< 0.001
Liver cancer*	54%	46%	< 0.001	53%	50%	47%	< 0.001	45%	59%	< 0.001
Colon cancer*	42%	35%	< 0.001	38%	41%	36%	< 0.001	33%	48%	< 0.001
Oral cancer*	31%	25%	< 0.001	30%	29%	24%	< 0.001	23%	36%	< 0.001
Skin cancer*	7%	5%	0.001	10%	5%	3%	< 0.001	5%	8%	< 0.001
Don’t know*	1%	2%	0.009	2%	2%	1%	0.040	2%	2%	0.598
None*	0%	0%	0.084	0%	0%	0%	0.013	0%	0%	0.178

* Only respondents selecting “cancer’ were asked to respond to the question about specific cancers, percentage represents proportion of all respondents

^a^ Weighted by age, gender, and education on country level based on the population distribution of EUROSTAT

^b^ Chi-squared test for differences in proportions was conducted to assess statistical significance. *P*-values below 0.05 are considered statistically significant

### Limitations

The study has certain limitations. Firstly, the study was conducted in only 14 countries and should therefore be considered as only a first attempt to systematically assess the knowledge of the link between alcohol use and cancer using the same survey across Europe. Although the included countries generally represent the various drinking patterns commonly found in Europe as well as in the different sub-regions, the study does not claim representativeness and was not conceptualized as a representative survey. Representativeness would involve probabilistic sampling methods with an inclusive sampling framework and no response biases. However, this does not seem possible in the field of alcohol research since the existing population-level surveys—which are typically household surveys—systematically under-report alcohol intake when compared to country sale statistics. This is due to the usual biases of self-report as well as a systematic exclusion of the heaviest drinkers in the sampling frames, including people who reside in treatment facilities, jails, or prisons, or who are experiencing homelessness [11]. Moreover, the sampling strategy included current drinkers only (i.e., people who have consumed alcohol in the past 12 months), and abstainers may differ in sociodemographic characteristics as well as in their alcohol-related knowledge. Therefore, the results on knowledge presented here should be interpreted with care, especially when compared to the reported-knowledge percentages in the mentioned reviews, which were more diverse in their sampling frames [5].

## Conclusions

This online survey assessed knowledge of alcohol-attributable conditions and cancer types across 14 EU countries and different sociodemographic groups for almost 20,000 participants, who were all current drinkers. Participants were well informed about the link between alcohol use and liver disease but lacked knowledge about the link between alcohol use and other NCDs, including liver cancer. Over 40% of participants did not recognize cancer as an alcohol-attributable condition, with significant variations seen between countries and population sub-groups. There might be various reasons for the observed stark differences in knowledge of alcohol-attributable diseases across European countries, ranging from differences in health education systems, presence or absence of public health campaigns related to alcohol use’s harms, media influence, social and cultural norms, as well as regulatory policies (for example, the level to which alcohol marketing is restricted in one country). The present results may therefore be used by countries to inform their public health or educational campaigns.

The findings also clearly highlight that knowledge surrounding alcohol use and specific cancers, particularly female breast cancer, was low, which reflects the findings of the recent review on existing studies [5]. Some participants mistakenly believed skin cancer to be alcohol-attributable, which suggests that there is a substantial proportion of individuals who might not have the relevant knowledge but simply follow the belief that “everything causes cancer” [12] and also indicates that the other knowledge percentages are likely to be overestimated.

Given the current scenario, in which the public and the alcohol consumers themselves are unaware of the causal relationship between alcohol and cancer, wider distribution and greater communication of this information is required, as well as information on how to mitigate the risk. Cancer-specific health warnings are already standard practice for tobacco products and should be considered for alcoholic beverages as well [13]. Additionally, public policy should encourage and support health professionals in discussing alcohol consumption with their patients, educating them about the various health risks, including cancer, and in assisting in behavioural changes [14]. Finally, population-wide strategies to reduce alcohol per capita consumption in European populations as a whole are needed, such as higher taxation of alcohol, for example [15]. Research suggests that individuals with increased knowledge of the causal link between alcohol consumption and cancer risk are more likely to support stricter alcohol regulations, including measures such as restrictions on sale hours or advertising [16]. Therefore, the findings indicate the importance of improving knowledge of the alcohol-cancer link as an integral part of broader prevention efforts in Europe.

### Electronic supplementary material

Below is the link to the electronic supplementary material.

Supplementary Material 1

Supplementary Material 2

Supplementary Material 3

Supplementary Material 4

## Data Availability

All data supporting the results in this article are included in the article itself or its supporting information. Any materials or databases generated in this study are available from the corresponding author upon reasonable request.

## Abbreviations

NCDs: Noncommunicable diseases
