# Supplementary material for: How much do Europeans know about the link between alcohol use and cancer? Results from an online survey in 14 countries
Source: BMC Res Notes. 2024 Feb 20;17:56. doi: 10.1186/s13104-024-06707-w (PMC10880362; doi:10.1186/s13104-024-06707-w)
Supplement: Supplementary file 1 — Supplementary Material 1 [file 13104_2024_6707_MOESM1_ESM.docx]

**Questionnaire**

Welcome to our survey!

***Section A: Introduction***

Dear participant:

This is a survey being conducted by the World Health Organization (WHO) Regional Oﬃce for Europe.

We are seeking the opinions and responses of current drinkers (individuals who have consumed at least 1 alcoholic drink in the past 12 months), who are 18 years of age and older. We will ask you to review some visual images and answer some questions using this online platform.

It will take 5-10 minutes of your time.

All your answers are anonymous and will be kept strictly conﬁdential. It will not be possible to link your individual responses to you in any way.

Your participation is entirely voluntary. You do not have to answer any question in this survey, nor do you have to complete the entire survey. If you choose to stop, your answers will be discarded and will not be used.

There is no direct beneﬁt from participating in this survey, however your answers will help us create images that can be widely distributed and are appropriate.

WHO may share the data and ﬁndings of this project, however no identifying information about participants will be presented.

If you agree to participate, click below, and continue.

 Yes  No

***Section B: This question is mandatory and assesses survey eligibility.***

1. Please indicate your age

- Under 18 years of age
- 18-20 years of age
- 21-24 years of age
- 25-29 years of age
- 30-34 years of age
- 35-39 years of age
- 40-44 years of age
- 45-49 years of age
- 50-54 years of age
- 55-59 years of age
- 60-64 years of age
- 65-69 years of age
- 70-74 years of age
- 75-79 years of age
- 80+ years of age

***Section C: This question is mandatory and assesses survey eligibility.***

2. The next questions are about how often you drank alcohol in the past 12 months and how much you drank usually on the days when you drank.

How often did you have a drink containing alcohol in the past 12 months?

- Never Monthly or less
- 2-4 times per month
- 2-3 times per week
- 4+ times per week

***Section D:***

3. How many standard drinks of alcohol did you drink on a typical day when you were drinking in the past 12 months?

- 1-2
- 3-4
- 5-6
- 7-9
- 10 +

4. How often have you had 6 or more standard drinks of alcohol on a single occasion in the past 12 months?

- Never
- Less than monthly
- Monthly
- Weekly
- Daily or almost daily

5. From the options below, what type of alcoholic drink do you consume most often?

- Beer
- Wine
- Spirits/Liquor

6. In my peer group it is acceptable to consume alcohol on a regular basis.

| 1 - Strongly Disagree | 2 - Disagree | 3 - Neither disagree nor agree | 4 - Agree | 5 - Strongly Agree |
| --- | --- | --- | --- | --- |
|  |  |  |  |  |

7. Please answer the following questions:

|  | 1 - Strongly Disagree | 2 - Disagree | 3 - Neither disagree nor agree | 4 - Agree | 5 - Strongly Agree |
| --- | --- | --- | --- | --- | --- |
| 1. If I consume more alcohol, there is a greater risk of harm to my health. |  |  |  |  |  |
| 1. If I consume alcohol on a regular basis, I am at greater risk of getting cancer. |  |  |  |  |  |
| 1. I intend to reduce the number of standard drinks that I drink in the forthcoming month. |  |  |  |  |  |

8. Which of the following diseases and conditions does alcohol consumption increase the risk of? (select all that apply)

- Cancer
- Heart Disease
- Liver Disease
- Respiratory Disease
- I don't know (do not select other option)
- None *(do not select other option)*

*In case option “Cancer” in Q8 was selected:*

8.1. Which of the following cancers do you think alcohol increases the risk of? (select all that apply)

- Female breast cancer
- Liver cancer
- Colon cancer
- Skin cancer
- Oral cancer
- I don't know (do not select other option)
- None *(do not select other option)*

***INTERVENTION: Please examine the following label on the front of the container.***

9. On a scale of 1 to 5, how much attention did you pay to the label presented?

| 1 – No attention at all | 2 | 3 | 4 | 5 – A high level of attention |
| --- | --- | --- | --- | --- |
|  |  |  |  |  |

*Now that you have examined the label, please answer the following question:*

10.1. Which of the following diseases and conditions does alcohol consumption increase the risk of? (select all that apply)

- Cancer
- Heart Disease
- Liver Disease
- Respiratory Disease
- I don't know (do not select other option)
- None *(do not select other option)*

*In case option “Cancer” in Q10.1 was selected:*

10.2. Which of the following cancers do you think alcohol increases the risk of? (select all that apply)

- Female breast cancer
- Liver cancer
- Colon cancer
- Skin cancer
- Oral cancer
- I don't know (do not select other option)
- None *(do not select other option)*

11. Now that you have examined the label, please answer the following questions:

|  | 1 - Strongly Disagree | 2 - Disagree | 3 - Neither disagree nor agree | 4 - Agree | 5 - Strongly Agree |
| --- | --- | --- | --- | --- | --- |
| 1. If I consume more alcohol, there is a greater risk of harm to my health. |  |  |  |  |  |
| 1. If I consume alcohol on a regular basis, I am at greater risk of getting cancer. |  |  |  |  |  |
| 1. I intend to reduce the number of standard drinks that I drink in the forthcoming month. |  |  |  |  |  |

***Please re-examine the following label on the front of the container.***

12. Based on the label above, please answer the following questions:

|  | 1 - Strongly Disagree | 2 - Disagree | 3 - Neither disagree nor agree | 4 - Agree | 5 - Strongly Agree |
| --- | --- | --- | --- | --- | --- |
| 1. This label is relevant to me |  |  |  |  |  |
| 1. Seeing this label would prompt me to talk to my family and/or friends about the health risk associated with alcohol. |  |  |  |  |  |
| 1. This label provides all the information I would need as a consumer. |  |  |  |  |  |
| 1. This label is clear and easy to understand |  |  |  |  |  |
| 1. This label is acceptable for alcohol products |  |  |  |  |  |
| 1. I would try to avoid thinking about the label. |  |  |  |  |  |
| 1. Seeing this label on an alcohol product would deter me from purchasing that item. |  |  |  |  |  |
| 1. I would prefer to receive the health-related message on this label via QR code. |  |  |  |  |  |
| 1. If there was a QR code included on this label as a link to more information, I would scan it with my mobile phone. |  |  |  |  |  |

13. Please indicate your gender

- Male
- Female
- Other/Prefer not to answer.

14. How many people are permanently living in your household, including yourself? _______

15. Which of these categories best describes the type of place where your main residence is located?

- A village or a farm
- A small city or town (below 50,000 residents)
- A medium-size city (50,000 - 250,000 residents)
- A large city (more than 250,000, up to 1 million residents)
- A very large city (over 1 million residents)

16. What is the highest school qualiﬁcation you have completed?

- Less than high school (less than Secondary education)
- High school (completed Secondary education)
- Post-secondary degree or certificate (Third level education)

17. Please specify the country where you currently live.

- Lithuania
- Ireland
- Germany
- Spain
- Norway
- Sweden
- Latvia
- Slovenia
- Iceland
- Estonia
- Belgium
- Austria
- Bulgaria
- Croatia
- Cyprus
- Czechia/Czech Republic
- Denmark
- Finland
- France
- Greece
- Hungary
- Italy
- Luxembourg
- Malta
- Netherlands
- Poland
- Romania
- Slovakia
- Portugal
- Ukraine

18. Do you have a migratory background*?

**A person with a migratory background is someone who has:*

*(a) migrated into their present country of residence; and / or*

*(b) previously had a different nationality from their present country of residence; and / or*

*(c) at least one of their parents previously entered their present country of residence as a migrant*

 Yes  No

19. Please specify your monthly net household income.

- Up to €499
- €500 to €999
- €1,000 to €1,499
- €1,500 to €1,999
- €2,000 to €2,499
- €2,500 to €2,999
- €3,000 to €3,499
- €3,500+

20. What device did you complete this survey on?

 Computer  Phone or tablet

--

Thank you for participating your responses have been saved.

Below is more information about the study.

*Study Title:* Multi-country effects of alcohol labels on alcohol-related knowledge and risk perceptions

*Lead research organization:* World Health Organization, Regional Office

for Europe

*Sponsor:* World Health Organization and European Commission

*Funder(s):* World Health Organization and European Commission

Thank you for completing the study, we appreciate your participation. We will now outline the main purpose and hypotheses of the study.

You were shown 1 out of 6 possible alcohol labels, each with varying amounts of information. You would have either viewed a plain label, one that encourages people to drink responsibly, one that indicates that alcohol is harmful to health, or one of 3 versions that says alcohol causes breast and colon cancer (with a pictogram, text only, or a graphical image). At the beginning of the study, we had to be vague about the details in order to reduce the bias introduced through anticipation of effects. Since we wanted to know if labels can spread awareness of the risk of cancer and alcohol, we did not want to give that information to participants at the beginning of the study.

We apologize for keeping this information from you; however it was important to do so to truly test the effect of the alcohol warning labels. As well, to test the level of attention paid to the labels, we also recorded the amount of time that you spend looking at the label. We apologize for not providing all the exact details of the study in the consent form and withholding the information that we recorded the time spent looking at the alcohol label.

We really appreciate your participation and hope that this has been an interesting experience for you.

If you have questions about this study, you can talk to the technical officer who is in charge of the study at WHO Regional Office for Europe. That person is Maria Neufeld.

Maria Neufeld

[neufeldm@who.int](mailto:neufeldm@who.int)
