# Supplementary material for: How much do Europeans know about the link between alcohol use and cancer? Results from an online survey in 14 countries
Source: BMC Res Notes. 2024 Feb 20;17:56. doi: 10.1186/s13104-024-06707-w (PMC10880362; doi:10.1186/s13104-024-06707-w)
Supplement: Supplementary file 2 — Supplementary Material 2 [file 13104_2024_6707_MOESM2_ESM.docx]

Table A2: Proportion of respondents selecting the condition where alcohol consumption increases the risk of, by sex, age and education, observed data

| All countries, N=19601 | Gender | | | Age | | | | Education | | |
| --- | --- | --- | --- | --- | --- | --- | --- | --- | --- | --- |
|  | Women | Men | p-value^a^ | 18-34 | 35-54 | 55+ | p-value^a^ | Secondary or less | Tertiary | p-value^a^ |
| N | 10007 | 9594 | - | 11851 | 5026 | 2724 | - | 9123 | 10478 | - |
| **Cancer** | 59% | 55% | <0.001 | 57% | 59% | 54% | <0.001 | 50% | 63% | <0.001 |
| **Heart disease** | 75% | 70% | <0.001 | 76% | 70% | 62% | <0.001 | 70% | 75% | <0.001 |
| **Liver disease** | 94% | 88% | <0.001 | 90% | 93% | 90% | <0.001 | 90% | 92% | <0.001 |
| **Respiratory disease** | 14% | 12% | <0.001 | 15% | 11% | 9% | <0.001 | 12% | 15% | <0.001 |
| **Don’t know** | 3% | 6% | <0.001 | 5% | 4% | 5% | 0.001 | 6% | 4% | <0.001 |
| **None** | 0% | 2% | <0.001 | 1% | 1% | 2% | <0.001 | 1% | 1% | 0.508 |
| *Female breast cancer** | 21% | 13% | <0.001 | 16% | 20% | 17% | <0.001 | 12% | 22% | <0.001 |
| *Liver cancer** | 57% | 52% | <0.001 | 55% | 56% | 51% | <0.001 | 47% | 61% | <0.001 |
| *Colon cancer** | 43% | 40% | <0.001 | 40% | 45% | 40% | <0.001 | 34% | 48% | <0.001 |
| *Oral cancer** | 33% | 30% | <0.001 | 32% | 32% | 27% | <0.001 | 26% | 36% | <0.001 |
| *Skin cancer** | 8% | 8% | 0.776 | 10% | 6% | 3% | <0.001 | 7% | 9% | <0.001 |
| *Don’t know** | 2% | 2% | 0.007 | 2% | 2% | 1% | 0.251 | 2% | 2% | 0.001 |
| *None** | 0% | 0% | 0.971 | 0% | 0% | 0% | 0.448 | 0% | 0% | 0.904 |

* Only respondents selecting “cancer’ were asked to respond to the question about specific cancers, percentage represents proportion of all respondents

^a^ Chi-squared test for differences in proportions was conducted to assess statistical significance. P-values below 0.05 are considered statistically significant
