# Supplementary material for: How much do Europeans know about the link between alcohol use and cancer? Results from an online survey in 14 countries
Source: BMC Res Notes. 2024 Feb 20;17:56. doi: 10.1186/s13104-024-06707-w (PMC10880362; doi:10.1186/s13104-024-06707-w)
Supplement: Supplementary file 3 — Supplementary Material 3 [file 13104_2024_6707_MOESM3_ESM.docx]

Table A3: Adjusted odds ratios (OR) and respective 95% confidence intervals (95%CI) of selecting the condition where alcohol consumption increases the risk, obtained through multivariate regression analysis, stratified by age group.

|  | **Cancer** | **Heart disease** | **Liver disease** | **Respiratory disease** | **Female breast cancer*** | **Liver cancer*** | **Colon cancer*** | **Oral cancer*** | **Skin cancer*** |
| --- | --- | --- | --- | --- | --- | --- | --- | --- | --- |
|  | OR (95%CI) | OR (95%CI) | OR (95%CI) | OR (95%CI) | OR (95%CI) | OR (95%CI) | OR (95%CI) | OR (95%CI) | OR (95%CI) |
| **Age 18-34** |  |  |  |  |  |  |  |  |  |
| *Gender* |  |  |  |  |  |  |  |  |  |
| Men | ref | ref | ref | ref | ref | ref | ref | ref | ref |
| Women | 1.15 (1.03, 1.27) | 1.33 (1.18, 1.50) | 1.75 (1.48, 2.09) | 1.26 (1.08, 1.46) | 1.42 (1.23, 1.65) | 1.17 (1.06, 1.30) | 1.14 (1.02, 1.27) | 1.13 (1.01, 1.27) | 1.11 (0.93, 1.33) |
| *Education* |  |  |  |  |  |  |  |  |  |
| Secondary or less | ref | ref | ref | ref | ref | ref | ref | ref | ref |
| Tertiary | 1.56 (1.40, 1.74) | 1.45 (1.28, 1.65) | 1.50 (1.25, 1.81) | 1.28 (1.10, 1.49) | 1.74 (1.50, 2.02) | 1.54 (1.38, 1.72) | 1.77 (1.58, 1.98) | 1.52 (1.35, 1.71) | 1.37 (1.14, 1.63) |
| **Age 35-54** |  |  |  |  |  |  |  |  |  |
| *Gender* |  |  |  |  |  |  |  |  |  |
| Men | ref | ref | ref | ref | ref | ref | ref | ref | ref |
| Women | 1.30 (1.19, 1.43) | 1.39 (1.26, 1.53) | 1.97 (1.67, 2.32) | 1.19 (1.04, 1.38) | 2.52 (2.21, 2.87) | 1.34 (1.22, 1.47) | 1.19 (1.08, 1.30) | 1.36 (1.23, 1.50) | 1.20 (0.99, 1.46) |
| *Education* |  |  |  |  |  |  |  |  |  |
| Secondary or less | ref | ref | ref | ref | ref | ref | ref | ref | ref |
| Tertiary | 2.30 (2.09, 2.52) | 1.51 (1.37, 1.66) | 2.24 (1.88, 2.68) | 1.37 (1.19, 1.58) | 2.97 (2.61, 3.37) | 2.23 (2.03, 2.44) | 2.17 (1.98, 2.38) | 2.23 (2.02, 2.46) | 1.79 (1.47, 2.18) |
| **Age 55+** |  |  |  |  |  |  |  |  |  |
| *Gender* |  |  |  |  |  |  |  |  |  |
| Men | ref | ref | ref | ref | ref | ref | ref | ref | ref |
| Women | 1.46 (1.32, 1.62) | 1.30 (1.17, 1.45) | 2.67 (2.24, 3.21) | 0.94 (0.79, 1.13) | 2.45 (2.10, 2.87) | 1.46 (1.31, 1.62) | 1.53 (1.37, 1.70) | 1.46 (1.30, 1.65) | 1.25 (0.92, 1.71) |
| *Education* |  |  |  |  |  |  |  |  |  |
| Secondary or less | ref | ref | ref | ref | ref | ref | ref | ref | ref |
| Tertiary | 1.51 (1.35, 1.69) | 1.42 (1.27, 1.59) | 1.38 (1.15, 1.66) | 1.07 (0.88, 1.29) | 2.54 (2.18, 2.95) | 1.49 (1.33, 1.66) | 1.51 (1.35, 1.69) | 1.88 (1.66, 2.13) | 1.48 (1.08, 2.02) |

* Only respondents selecting “cancer” were asked to respond to the question about specific cancers, percentage represents proportion of all respondents.
