# Supplementary material for: How much do Europeans know about the link between alcohol use and cancer? Results from an online survey in 14 countries
Source: BMC Res Notes. 2024 Feb 20;17:56. doi: 10.1186/s13104-024-06707-w (PMC10880362; doi:10.1186/s13104-024-06707-w)
Supplement: Supplementary file 4 — Supplementary Material 4 [file 13104_2024_6707_MOESM4_ESM.docx]

Table A1: Proportion of respondents selecting the condition where alcohol consumption increases the risk of, total and by country, observed data

| All countries, N=19601 | **Total** | Austria | Belgium | Estonia | France | Germany | Ireland | Latvia | Lithuania | Netherlands | Norway | Portugal | Slovenia | Spain | Sweden | p-value^a^ |
| --- | --- | --- | --- | --- | --- | --- | --- | --- | --- | --- | --- | --- | --- | --- | --- | --- |
| N | **19601** | 1356 | 885 | 969 | 1876 | 2565 | 923 | 1386 | 511 | 758 | 1126 | 2345 | 1144 | 2826 | 931 | - |
| **Cancer** | **57%** | 58% | 60% | 48% | 75% | 67% | 59% | 35% | 54% | 61% | 48% | 53% | 44% | 62% | 56% | <0.001 |
| **Heart disease** | **73%** | 76% | 77% | 78% | 87% | 83% | 69% | 76% | 80% | 73% | 59% | 57% | 63% | 71% | 69% | <0.001 |
| **Liver disease** | **91%** | 96% | 90% | 94% | 93% | 97% | 93% | 92% | 94% | 92% | 91% | 84% | 94% | 85% | 93% | <0.001 |
| **Respiratory disease** | **13%** | 10% | 11% | 16% | 18% | 16% | 19% | 10% | 14% | 13% | 7% | 11% | 9% | 18% | 7% | <0.001 |
| **Don’t know** | **5%** | 2% | 5% | 4% | 3% | 2% | 4% | 5% | 3% | 6% | 7% | 7% | 4% | 7% | 3% | <0.001 |
| **None** | **1%** | 1% | 1% | 1% | 1% | 1% | 0% | 1% | 2% | 1% | 1% | 3% | 1% | 1% | 2% | <0.001 |
| *Female breast cancer** | **17%** | 16% | 18% | 15% | 29% | 23% | 27% | 11% | 18% | 20% | 10% | 9% | 8% | 15% | 18% | <0.001 |
| *Liver cancer** | **55%** | 55% | 57% | 45% | 73% | 63% | 57% | 33% | 52% | 57% | 43% | 52% | 42% | 60% | 50% | <0.001 |
| *Colon cancer** | **41%** | 44% | 45% | 31% | 58% | 53% | 41% | 25% | 38% | 45% | 33% | 30% | 32% | 44% | 41% | <0.001 |
| *Oral cancer** | **31%** | 31% | 34% | 23% | 51% | 43% | 36% | 15% | 26% | 31% | 16% | 26% | 29% | 33% | 22% | <0.001 |
| *Skin cancer** | **8%** | 8% | 8% | 8% | 16% | 14% | 8% | 6% | 8% | 8% | 3% | 4% | 3% | 9% | 3% | <0.001 |
| *Don’t know** | **2%** | 2% | 2% | 2% | 2% | 2% | 1% | 1% | 1% | 3% | 3% | 1% | 2% | 1% | 4% | <0.001 |
| *None** | **0%** | 0% | 0% | 0% | 0% | 0% | 0% | 0% | 0% | 0% | 0% | 0% | 0% | 0% | 0% | 0.574 |

* Only respondents selecting “cancer’ were asked to respond to the question about specific cancers, percentage represents proportion of all respondents

^a^ Chi-squared test for differences in proportions was conducted to assess statistical significance. P-values below 0.05 are considered statistically significant
